# Sus-Pense

**DOI:** 10.3201/eid1603.091250

**Published:** 2010-03

**Authors:** R.L. Bernstein

**Affiliations:** New Mexico State University, Las Cruces, New Mexico, USA

**Keywords:** swine, pigs, H1N1, pandemic (H1N1) 2009, influenza, another dimension

Don’t call it swine flu:
if you dine on pork, you
can’t catch the flu, as we know.
But the pork people say
some customers may
be confused by the name and just go.
Now look at the news:
around the world views
show people with a mask on their face.
Despite the name change,
folks who find H1N1 strange
still want to keep swine in their place.
For me it is clear:
I don’t have the fear
of swallowing bacon-wrapped figs.
While others may hurry
to wear masks, I don’t worry.
I really don’t plan to kiss pigs.

